# IgM and IgG Immunoreactivity of SARS-CoV-2 Recombinant M Protein

**DOI:** 10.3390/ijms22094951

**Published:** 2021-05-07

**Authors:** Zorana Lopandić, Isidora Protić-Rosić, Aleksandra Todorović, Sofija Glamočlija, Marija Gnjatović, Danica Ćujic, Marija Gavrović-Jankulović

**Affiliations:** 1 Department of Biochemistry, Faculty of Chemistry, University of Belgrade, 11000 Belgrade, Serbia; lopandic@chem.bg.ac.rs (Z.L.); proticrosic@chem.bg.ac.rs (I.P.-R.); 2 Institute for the Application of Nuclear Energy, University of Belgrade, 11000 Beograde, Serbia; aleksandra.todorovic@inep.co.rs (A.T.); sofija.glamoclija@inep.co.rs (S.G.); marijad@inep.co.rs (M.G.); danicac@inep.co.rs (D.Ć.)

**Keywords:** SARS-CoV-2 membrane protein, COVID-19, IgM reactivity, IgG reactivity, antigenicity, ELISA

## Abstract

Diagnostic evaluation of specific antibodies against the SARS-CoV-2 virus is mainly based on spike (S) and nucleocapsid (N) proteins. Despite the critical functions in virus infection and contribution to the pattern of immunodominance in COVID-19, exploitation of the most abundant membrane (M) protein in the SARS-CoV-2 serology tests is minimal. This study investigated the recombinant M protein’s immunoreactivity with the sera from COVID-19 convalescents. In silico designed protein was created from the outer N-terminal part (19 aa) and internal C-terminal tail (101–222 aa) of the M protein (YP_009724393.1) and was recombinantly produced and purified. The designed M protein (16,498.74 Da, p*I* 8.79) revealed both IgM and IgG reactivity with serum samples from COVID-19 convalescents in Western blot. In ELISA, more than 93% (28/30) of COVID-19 sera were positive for IgM detection, and more than 96% (29/30) were positive for specific IgG detection to M protein. Based on the capacity to provoke an immune response and its strong antigenic properties, as shown here, and the fact that it is also involved in the virion entry into host cells, the M protein of the SARS-CoV-2 virus as a good antigen has the potential in diagnostic purposes and vaccine design.

## 1. Introduction

An outbreak of coronavirus in Wuhan was reported by the World Health Organization (WHO) country office in China in December 2019 [1]. The identified infectious agent, named the SARS-CoV-2 virus, rapidly spread worldwide and causing a global COVID-19 pandemic, with the severe acute respiratory syndrome (SARS) as the most severe clinical manifestation of the disease [2]. Soon after, the genome of the enveloped SARS-CoV-2 virus was sequenced as a positive sense, single-stranded RNA, with the size of 29.8–30 kb [3]. At the 5′ terminal, two-thirds of the genome consists of two open reading frames (ORFs), ORF1 and ORF2, encoding the RNA polymerase and other non-structural proteins of the virus [4]. At the 3′ terminal, four major structural proteins are encoded in the following order: the spike (S) glycoprotein (transcribed into 1273 aa), envelope (E) protein (76 aa), membrane (M) protein (222 aa), and nucleocapsid (N) protein (419 aa), all of which are involved in the production of the structurally complete viral particle [5].

By 9 March 2021 the number of infected persons reached more than 118,000,000 and more than 2,600,000 death cases (https://coronavirus.jhu.edu/map.html, accessed on 9 March 2021). The course of COVID-19 varies in clinical manifestations and disease severity. The wide range of clinical manifestations is probably due to the virus infecting almost every human cell [6]. Therefore, understanding viral structure and interaction with host cells are important for developing strategies against the virus [7]. To decrease viral spreading and control pandemics, prophylactic, therapeutic, and diagnostic solutions are needed. So far, the most attention was given to the S and N proteins of the SARS-CoV-2 virus. This study focused on M protein, the most abundant structural protein of the SARS-CoV-2 which performs a variety of critical functions in the virus infection cycle. Due to its abundance, it may be one of the key components for virion assembly and morphogenesis [8]. It assists to assemble all other structural proteins (S, E, N) and participates in the budding process [9]. High-resolution structures of structural SARS-CoV-2 proteins have become available in the Protein Data Bank for S (6XEY), E (7K3G), and N (N-terminal domain 7CDZ, C-terminal domain 7CE0) protein; however, the structure of M protein has been only in silico predicted [10,11]. In addition, there is not much information about the immunoreactivity of SARS-CoV-2 M protein.

To provide timely medical help to the COVID-19 patient, accurate diagnosis is of utmost importance. At the moment, there are two approaches in COVID-19 laboratory diagnostics: detection of virus presence or detection of antibody response to viral infection. PCR is assumed as a gold standard for COVID-19 confirmation, although the reliability of results is highly dependent on the time when the test is performed [12]. Antibodies specific for the important SARS-CoV-2 antigens, the S glycoprotein which binds to the human ACE2 receptor for viral entry, and the N protein necessary for viral replication, have been detected in actively infected and convalescent patients with the mild disease [13,14].

Enormous efforts have been focused on developing effective and safe drugs and vaccines against SARS-CoV-2. Various therapeutic options, including herbal medicine, have been explored to fight against the SARS-CoV-2 virus [15,16]. Also, by 24 September 2020, the SARS-CoV-2 vaccine landscape included 43 candidates based on inactivated vaccines, nucleic acid vaccines, vector vaccines, and protein vaccines which have been tested in clinical trials [16,17], with several that have already been authorized for vaccination. By 18 April 2021 a guidance document, listing current COVID-19 vaccine candidates undergoing assessment for WHO Emergency Use Listing (EUL) and prequalification (PQ), is available on the following web address: https://www.who.int/publications/m/item/draft-landscape-of-covid-19-candidate-vaccines, accessed on 18 April 2021. Understanding the development of adaptive immunity to the SARS-CoV-2 virus is essential for vaccine development and setting respective pandemic control measures. Mapping the B-cell epitopes on the COVID-19 antigens, which recognize and bind specific antibodies, is valuable for the development of diagnostic tests, synthetic vaccines, and novel immunotherapeutics [18]. Analysis of circulating SARS-CoV-2-specific CD8+ and CD4+ T cells in blood samples from COVID-19 patients revealed the immunodominance pattern for M, S, and N proteins, each recognized by 100% of COVID-19 cases studied. In the context of CD8+ cells reactivity in COVID-19 blood samples, M protein was identified as strong as the spike protein [19].

Serology test detection of specific antibodies against the SARS-CoV-2 virus is mainly based on S and N proteins or a combination thereof [20], although other viral proteins also provoke an immune response. At this moment, data regarding the use of M protein in serology tests for COVID-19 are minimal. This study investigated the recombinant M protein’s immunoreactivity with IgM and IgG antibodies in sera of COVID-19 convalescents.

## 2. Results

### 2.1. Production and Purification of the Recombinant M Protein

**Figure 1:**
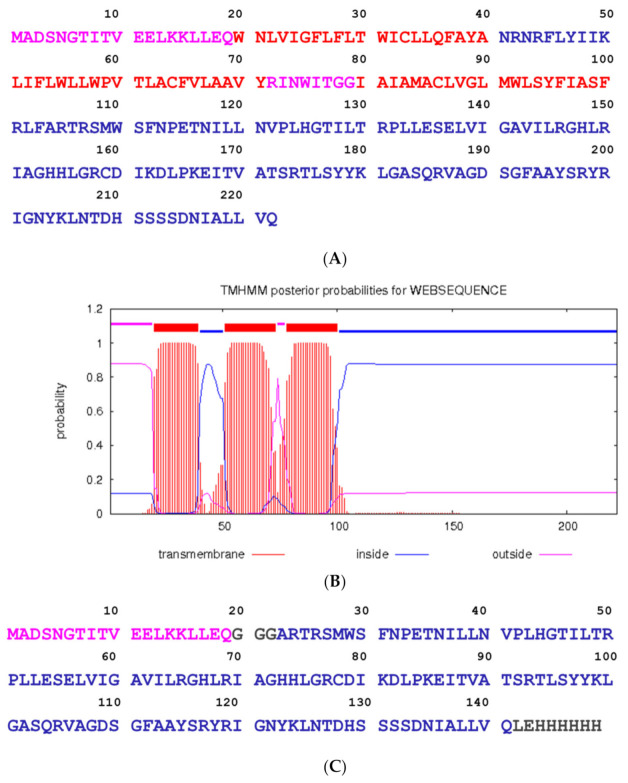

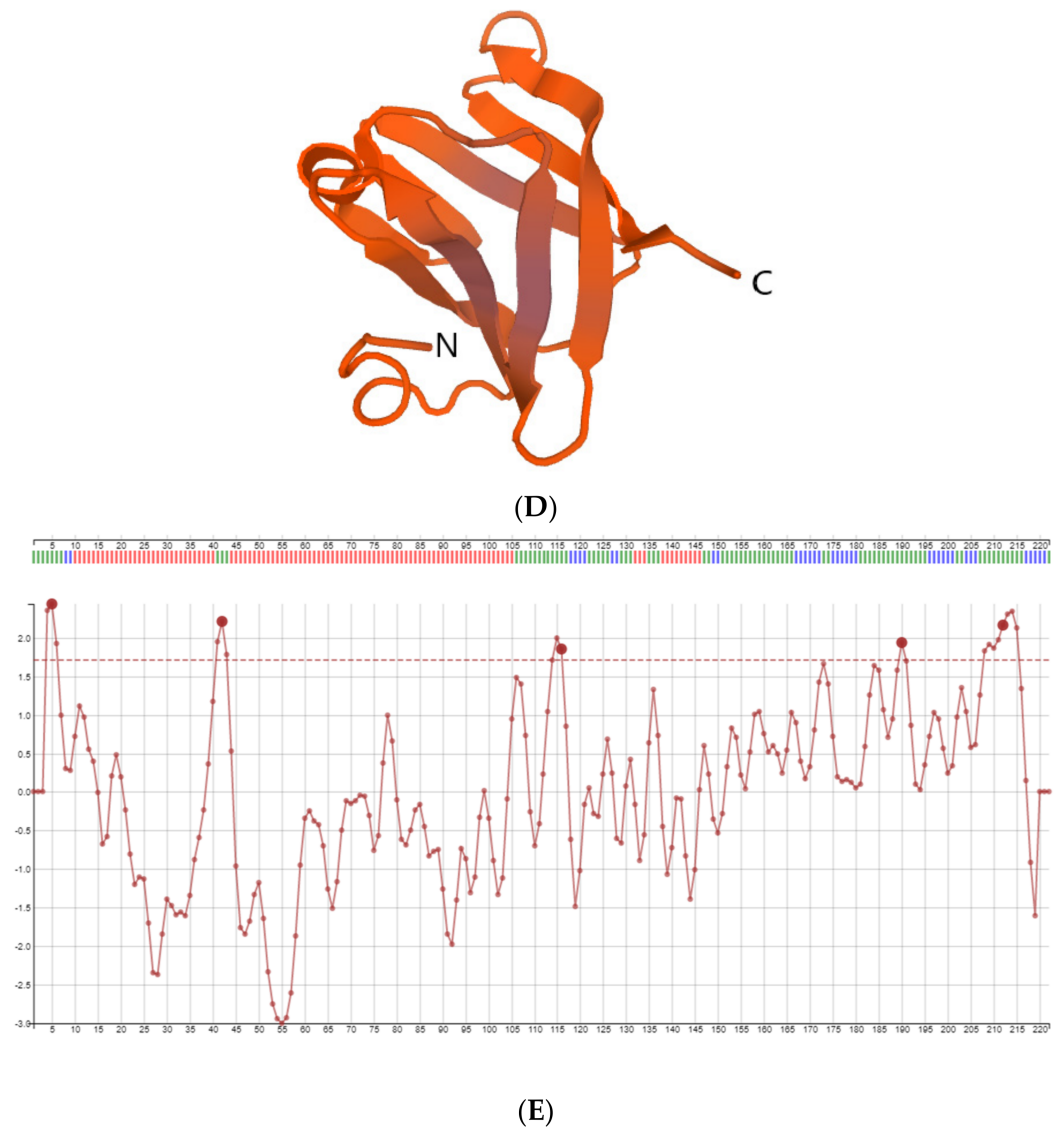
Membrane (M) protein of SARS-CoV-2: (**A**) amino acid sequence NCBI YP_009724393.1; (**B**) Prediction of transmembrane regions in the primary structure of M protein in http://www.cbs.dtu.dk/services/TMHMM (accessed on 9 March 2021). (**C**) amino acid sequence of the recombinant M protein with 6His tag; Position of amino acids in the virion particle: amino acids exposed on the virion surface are shown in pink, amino acids inside the virion are shown in blue, amino acids in the transmembrane regions are shown red, and amino acids derived from the cloning strategy are shown in grey. (**D**) Structure of the M protein fragment obtained in SWISS-MODEL (25–121 aa). (**E**) Five B cell epitopes predicted in the SARS CoV-2 M protein in BEPITOP: 1MADSNGTITVEE, 35LQFAYANRNRFLYII, 109MWSFNPETNILLNV, 184SQRVAGDSGFAAY, 202GNYKLNTDHSSSSDNIALLV.

**Figure 2:**
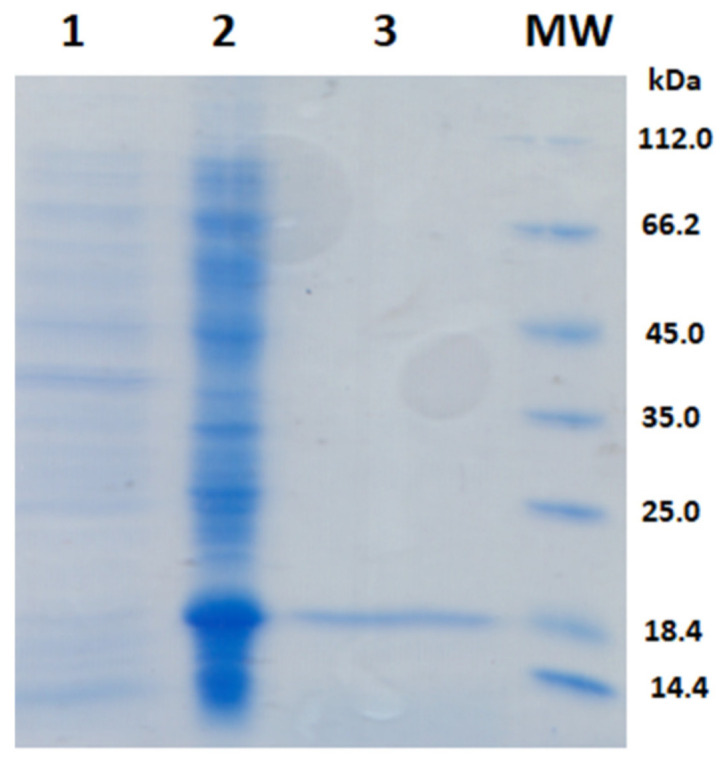
SDS-PAGE of the recombinant M protein: Samples were separated on 14% SAS-PAGE, after which the gel was stained with CBB R 250. Lines: MW) molecular weight markers; (1) cell lysate before the addition of IPTG, (2) cell lysate after 6 h of expression, (3) purified M protein after IMAC.

Analysis of the primary structure of the M protein SARS CoV-2 (NCBI YP_009724393.1) by bioinformatics tools (http://www.cbs.dtu.dk/services/TMHMM/, accessed on 25 April 2021) revealed the N-terminal region (1–19 amino acids), three distinct transmembrane regions with helical structure: (TMI 20–39 amino acids, TMII 51–73 amino acids, TMIII 78–100 amino acids), and C-terminal region (101–222 amino acids) (Figure 1). Five continuous protein epitopes were predicted in the program BEPITOP: ^1^MADSNGTITVEE, ^35^LQFAYANRNRFLYII, ^109^MWSFNPETNILLNV, ^184^SQRVAGDSGFAAY, ^202^GNYKLNTDHSSSSDNIALLV. Therefore, the cloning strategy employed the first 19 amino acids on the N-terminal and amino acids 104–222 at the C-terminal. The linker GGG was inserted between these two segments. Restriction site *Xho*I adds amino acids LE, and the addition of a 6His tag to the C terminus enhances protein purification. The 149 amino acids M protein’s theoretical molecular mass was 16,498.74 Da, and p*I* of 8.79. Designed M protein was produced in *E. coli*. SDS-PAGE analysis of purified M protein by the IMAC is shown in Figure 2. The gel stained with CBB revealed a protein band of about 17 kDa.

### 2.2. Immunoreactivity of the Recombinant SARS-CoV-2 M Protein in Western Blot

**Figure 3:**
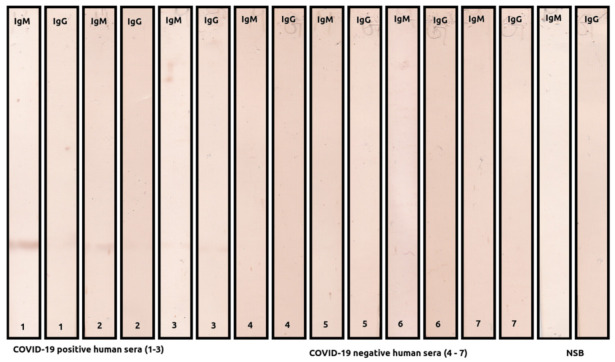
Representative Western blots showing the reaction of sera from COVID-19 subjects (samples 1−3) and healthy individuals (samples 4−7); NSB–nonspecific binding of anti-human IgM and IgG antibodies.

Immunoreactivity of the recombinant SARS-CoV-2 M protein was tested in Western blot (Figure 3). Samples from COVID-19 convalescents showed both IgM and IgG reactivity against M protein. A single band was detected, demonstrating good purity and specificity of produced M protein. In contrast, sera samples from non-COVID-19 subjects did not react with the recombinant M protein. Nonspecific binding of human IgM and IgG to M protein was not observed.

### 2.3. Immunoreactivity of the SARS-CoV-2 M Protein in ELISA

**Figure 4:**
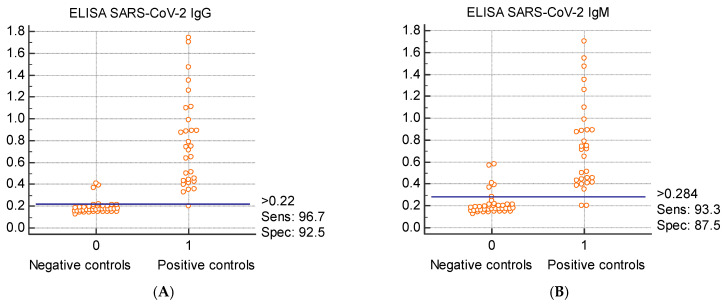

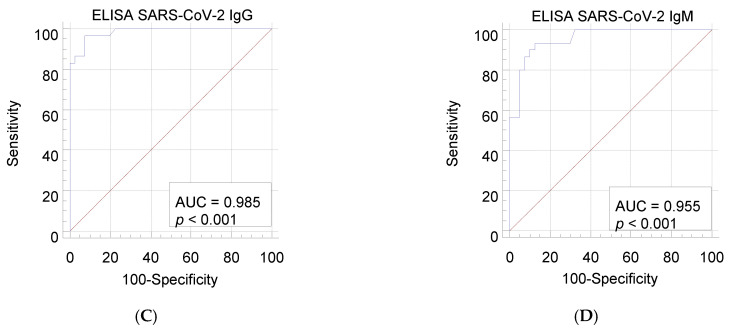
Distribution of the OD values obtained from 40 SARS-CoV-2 negative and 30 SARS-CoV-2 positive human sera samples using recombinant M protein-based SARS-CoV-2 IgG (**A**) and IgM (**B**) indirect ELISA. The ROC curve built for 40 SARS-CoV-2 negative and 30 SARS-CoV-2 positive human sera analyzed by SARS-CoV-2 IgG (**C**) and IgM (**D**) ELISA based on M protein.

For optimization of the new assay based on SARS-CoV-2 M protein, 10 SARS-CoV-2 positive and 10 negative sera were used for the ELISA SARS-CoV-2 IgG and IgM testing. The widest range in OD values for negative and positive sera for both classes of antibodies was observed when using M protein as the coating antigen at a final concentration of 2 μg/mL (OD 0.054−1.750 for IgG, OD 0.067−1.547 for IgM). The optimal dilution of the test sera was 1:50. Using this optimal dilution of coating protein and sera, the optimal dilution of the HRP conjugated anti-human IgG was found to be 1:5000, and anti-human IgM was 1:8000. The distribution of the OD values obtained from 40 SARS-CoV-2 negative and 30 SARS-CoV-2 positive human sera using IgG and IgM ELISA is presented in Figure 4A,B. According to the receiver operating characteristic (ROC) curve analysis, the best cut-off value for IgG detection was 0.220 OD (Figure 4C), and using this value, sensitivity was 96.7%, and specificity was 92.5%.

Based on the above-mentioned criteria, 37 samples from the 40 negative control sera were negative, and 29 samples from the 30 positive control sera were positive (OD 0.220−1.75) in the SARS-CoV-2 IgG ELISA. The area under the curve (AUC) was 0.985, which suggests that defined cut-offs are suitable for interpreting ELISA results and attainment of high precision in identifying the presence or absence of anti-SARS-CoV-2 specific IgG antibodies in human sera (the maximum AUC is 1, which corresponds to a perfect classifier, whereas large AUC values indicate better classifier performance).

The best cut-off value for IgM detection (Figure 4D) was 0.284, with a sensitivity of 93.3% and specificity of 87.5%. In detecting M protein-specific IgM antibodies, 35 sera from the group of negative control sera (*n* = 40) were negative in the assay, and 28 sera from 30 SARS-CoV-2 positive samples were positive in the SARS-CoV-2 IgM ELISA assay. The AUC was 0.955, indicating an acceptable degree of accuracy in detecting SARS-CoV-2 specific IgM antibodies in an assay based on M protein. The test performance characteristics of IgM and IgG ELISA tests are summarized in Table 1, showing that ELISA tests based on M protein could discriminate very well between COVID-19 subjects and healthy controls.

## 3. Discussion

The majority of the serological tests for the detection of specific antibodies in serum of COVID-19 patients on the market are based on the SARS-CoV-2 spike (S) glycoprotein and the nucleocapsid (N) protein. The S glycoprotein protrudes from the virus envelope and consists of the S1 subunit, which contains the receptor-binding domain, and the S2 subunit, which is involved in host cell membrane fusion. The nucleocapsid protein is about three times smaller than S glycoprotein and lacks glycosylation sites. Both S and N proteins are immunogenic and are suitable for application in diagnostic assay [21]. Besides diagnostic significance, immunoreactivity of viral proteins is also significant for vaccine design. Currently, vaccine development is based either on attenuated SARS-CoV-2 viral particle or S protein as dominant immunogen [22]. SARS-CoV M protein provoked both humoral and cellular immune responses [23]. Antibodies directed to M protein were detected ten days post-onset in over 95% of the SARS-CoV convalescent patients (*n* = 58) [24]. The primary structure of the SARS-CoV-2 M protein (YP_009724393.1) has shown 91% (201/222) of sequence identities and 96% (214/222) of sequence similarities to SARS-CoV M protein (UniProtKB-P59596) [25] and only 42% (86/203) of sequence identity and 61% (124/203) of the sequence similarity with the MERS-CoV M protein (UniProtKB-T2BB40). In silico analysis of M protein’s primary structure revealed the transmembrane helix topology, with the N- and C-terminal parts being exposed outside and inside the virus particle, respectively. The predominant secondary structures of the long internal part of the M protein seem to be β-strands with coiled coil. Thomas reported homology of the M protein with the prokaryotic sugar transport protein SemiSWEET, which might contribute to the rapid proliferation, replication, and immune evasion of the SARS-CoV-2 virus [11]. The three-dimensional model of the M protein fragment has been predicted in SWISS-MODEL and it shared 15.6% of sequence identity with the Cryo-EM structure of SARS-CoV-2 ORF3a protein (SMTL ID: 7kjr.1.A).

Grifoni et al. reported on the pattern of immunodominance in COVID-19 cases, revealing M, S, and N proteins as clearly co-dominant, by each, recognized by 100% of COVID-19 cases (*n* = 20) [19]. Our study aimed to investigate the immunological reactivity of recombinant SARS-CoV-2 M protein. Humoral immune response to M protein was tested using recombinant M protein as antigen for detection of specific IgM and IgG antibodies in COVID-19 convalescent patients in ELISA and Western blot. The results demonstrated that the M protein of SARS-CoV-2 has strong antigenic properties. M protein’s immunoreactivity was very comparable to S and N protein’s immunoreactivity when measured in ELISA. Immunodominant epitopes of the SARS-CoV virus are previously reported on both N- and C-terminus of M protein [24,26]. In this study, we choose regions of M protein that protrude outside and inside the virion, which bears four out of five in silico predicted continuous epitopes and demonstrated their IgM and IgG reactivity. More than 96% of COVID-19 sera were positive in the ELISA test for specific IgG detection, and more than 93% of COVID-19 sera were positive in the ELISA test for IgM detection.

Individual SARS-CoV-2 antigens revealed different performances to detect IgG and IgM [27]. De Assis et al. also suggested that different purification tags may significantly affect the antigen conformation and affect antibody binding. They indicated that combining additional antigens, such as the M protein, could contribute to the overall diagnostic performance.

Viral infection provokes the immune system to produce neutralizing antibodies against immunodominant virus epitopes. The main target for the induction of neutralizing antibodies is S protein that protrudes from viral particles and interacts with host cells’ receptors. Limited data are available regarding the functional properties of antibodies raised against the SARS-CoV-2 M protein. Neutralizing antibodies directed to M protein of SARS-CoV virus are described [28], while monoclonal antibodies to M protein also may demonstrate blocking activity [29]. The engagement of multiple antibodies through different protective mechanisms can effectively control virus infections [30]. Immunodominant epitopes on the M protein of the SARS-CoV were identified on the N-terminal region (residues 1 to 31) and the interior C-terminal region (residues 132 to 161), respectively [26]. Given the fact that M protein possesses strong antigenic properties, as shown here and that can provoke an immune response to the SARS-CoV-2 virus, as well as the fact that it is also involved in the entry of virus in host cells, there are good reasons for further study potential of M protein for diagnostic purposes either solely or in combination with other SARS-CoV-2 proteins. The fact that M protein is conserved among coronaviruses and less prone to mutations than S protein gives a justifiable reason for M protein to be a candidate for vaccine and diagnostic assay development [31].

## 4. Materials and Methods

### 4.1. Design and Production of the Recombinant M Protein SARS-CoV−2

For a suitable 3D template the SWISS-MODEL was employed. We used amino acid sequence of the M protein fragment and the 15.63% sequence identity was revealed by SARS-CoV-2 Protein ORF3a (PDB ID: 6XDC), which looks like cytoplasmic part of protein. In silico prediction of immunoreactivity for the SARS-CoV-2 M protein was done in the BEPITOPE program [32]. One continuous epitope was localized in the N-terminus (^1^MADSNGTITVEE) and three were distributed in the C terminus (^109^MWSFNPETNILLNV, ^184^SQRVAGDSGFAAY, ^202^GNYKLNTDHSSSSDNIALLV, 109−221) therefore the cloning strategy was to combine first 19 amino acids with amino acids 101−222 connected with GGG linker. The gene for the M protein (Gene ID 43740571), from SARS-CoV-2 without transmembrane regions was cloned in pET23b vector with restriction enzymes *Nde*I and *Xho*I (ThermoFisher Scientific, Wathman, MA, USA). Expression of the M protein was done in BL21-CodonPlus (DE3)-Ripl cells (Agilent Technologies Inc., La Jolla, CA, USA) in Luria-Bertani medium (LB medium) supplemented with 100 μg/L ampicillin (Carl Roth, Karlsruhe, Germany), 25 μg/L kanamycin (Carl Roth, Karlsruhe, Germany), 25 μg/L chloramphenicol (Carl Roth, Karlsruhe, Germany). A single colony containing pET-23b-M vector was inoculated in 5 mL of LB medium with antibiotics. The culture was left overnight (ON) at 37 °C in a shaking incubator (Bio San, Riga, Latvia). Five mL of the ON culture was inoculated in 1 L of sterile LB medium with appropriate antibiotics at 37 °C under constant shaking (250 rpm). When absorbance at 600 nm (OD600) reached 0.6, the temperature of the medium was lowered to 22 °C, and expression was induced using 1 mM isopropyl-β-D-1-thiogalactopyranoside (IPTG) (ThermoFisher Scientific, Waltham, MA, USA). Cells were grown at 22 °C ON under constant shaking (250 rpm). Cells were harvested by centrifugation (3000× *g*, 25 min at 4 °C, Eppendorf centrifuge 5430R, Hamburg, Germany), suspended in Lysis buffer (20 mM Tris-HCl (Merck KGaA, Darmstadt, Germany), 150 mM NaCl (Beta Hem, Belgrade, Serbia), 1% Triton-X-100 (Merck KGaA, Darmstadt, Germany), 10 mM dithiothreitol (Serva Electrophoresis GmbH, Heidelberg, Germany), pH 7.8 and sonicated in an ice water bath (10 × 10 s at 30 W, Branson sonifier 150 (Branson Ultrasonic SA, Carouge, Switzerland). The M protein was solubilized from inclusion bodies in 6 M urea (Serva Electrophoresis GmbH, Heidelberg, Germany), and applied onto a metal immobilized affinity chromatography (IMAC, Talon^®^ Superflow™, Uppsala, Sweden) according to the manufacturer’s instructions. The bound fraction was eluted using 20 mM Tris-HCl, 150 mM NaCl, 6 M urea, 300 mM imidazole pH 7.8. After gel electrophoresis, eluted fractions were pulled and dialyzed against 20 mM Tris-HCl, 150 mM NaCl, 0.1% SDS (Serva Electrophoresis GmbH, Heidelberg, Germany) pH 7.8. The level of protein purity was analyzed by staining SDS-PA gel with CBB (Serva, Electrophoresis GmbH, Heidelberg, Germany).

### 4.2. Patient Serum Samples

The Medical Ethical Committee of the Institute for the Application of Nuclear Energy (INEP, Belgrade, Serbia) approved this study. All study participants gave written informed consent to use their serum samples for ELISA SARS-CoV-2 assay development. A total of 70 human serum samples were analyzed in ELISA. Blood samples for COVID-19 convalescents (*n* = 30) were taken at least four weeks after disease onset. COVID-19 infection was confirmed with the quantitative RT-PCR, and anti-SARS-CoV-2 antibodies in these sera were confirmed by ELISA SARS-CoV-2 IgG and IgM (INEP, Belgrade, Serbia). Sera samples collected from healthy persons (*n* = 40) before the COVID-19 outbreak in Serbia taken from the INEP sera sample bank were used as a negative control.

### 4.3. Detection of M Protein Specific IgM and IgG in Western Blot

Specific IgM and IgG for the SARS-CoV-2 membrane protein in COVID-19 patients sera were tested in Western blot. M protein was resolved on (4/14%) SDS-PAGE under reducing conditions (Mini Protean System, Bio-Rad, CA, USA). After semi-dry transfer to a nitrocellulose membrane (ThermoFisher Scientific, Waltham, MA, USA), the membrane was blocked in blocking buffer (1% BSA/10 mM PBS, 150 mM NaCl, 0.05% Tween-20, pH 7.2) for 1 h at room temperature. Randomly selected sera samples from COVID-19 convalescents and control subjects, previously tested in ELISA as described above (diluted 1:50 in blocking buffer) were individually incubated with membrane overnight on 4 °C. Membranes incubated in blocking buffer, omitting serum, were used for assessment of nonspecific binding. Incubation with sheep anti-human IgM/HRP or anti-human IgG/HRP conjugate (INEP, Belgrade, Serbia), diluted 1:1000 in blocking buffer, was carried out one hour at room temperature. Following each incubation step, membranes were washed in PBST. DAB staining was used (EnVision Detection Systems Peroxidase/DAB, DAKO, Santa Clara, CA, USA). Membranes were washed in water, dried, and scanned.

### 4.4. ELISA

Sera previously tested in the ELISA SARS-CoV-2 IgM and IgG INEP, Serbia (10 positive and 10 negative), were used to optimize the new ELISA based on recombinant M protein. The plates were coated with the recombinant M protein in different concentration (0.5 μg/mL, 1 μg/mL, 2 μg/mL, 3 μg/mL) in 0.1 M carbonate buffer pH 9.5 (100 μL/well). The plates were incubated at +4 °C for 18 h and washed three times with phosphate-buffered saline pH 7.6 (PBS) containing 0.05% Tween 20 (PBST). The antigen-coated plates were incubated with blocking solution (0.1 mg BSA in PBST, 100 μL/well) for 1 h at 37 °C. After washing, human sera diluted in blocking solution were added (1:50, 1:100, 1:200, 1:300, 1:400, 1:500; 100 μL/well) and plates were incubated for 1 h at 37 °C. After washing, the HRP-conjugated sheep anti-human IgM and anti-human IgG (INEP, Belgrade, Serbia) were diluted in blocking solution and added to the wells (1:1000, 1:2000, 1:5000, 1:8000; 100 μL/well) and then incubated for 1 h at 37 °C. After washing 4 times with washing buffer (PBST), the color was allowed to develop for 10 min with the chromogenic solution (3,3’,5,5’-tetramethylbenzidine (TMB) substrate, INEP, Serbia/hydrogen peroxide). After stopping the reaction with Stop solution (INEP, Belgrade, Serbia), optical densities were measured at 450 nm using an ELISA reader (Wallac Multilabel Counter 1420, Perkin Elmer, Milano, Italy).

### 4.5. Statistical Analysis

In total, 70 sera samples (40 negative and 30 positive) were used for preliminary ELISA assay validation. Statistical analyses were carried out using MedCalc version 10.4.0.0 (MedCalc Software, Ostend, Belgium) [33,34]. Sensitivity was defined as the proportion of correctly identified COVID-19 positive patients who were initially positive by RT-PCR SARS-CoV-2 determination in respiratory samples. Specificity was defined as the proportion of naive participants classified as positive as analyzed by ELISA SARS-CoV-2 M protein-specific antibodies.

## Figures and Tables

**Table 1:** M Protein-Based ELISA IgM and IgG Tests Performance Characteristics.

	ELISA IgM	ELISA IgG
Sensitivity (%)	93.3	96.7
Specificity (%)	87.5	92.5
Negative predictive value (%)	91.1	97.4
Positive predictive value (%)	84.8	90.6

## Data Availability

The data presented in this study are available on request from the corresponding author.
